# Positive selection at sites of chemosensory genes is associated with the recent divergence and local ecological adaptation in cactophilic *Drosophila*

**DOI:** 10.1186/s12862-018-1250-x

**Published:** 2018-09-20

**Authors:** Fernando Diaz, Carson W. Allan, Luciano M. Matzkin

**Affiliations:** 1 Department of Entomology, University of Arizona, Tucson, AZ 85721 USA; 2 Department of Ecology and Evolutionary Biology, University of Arizona, Tucson, AZ 85721 USA; 3 BIO5 Institute, University of Arizona, Tucson, AZ 85721 USA

**Keywords:** Odorant receptor, Gustatory receptor, Population genetics, Molecular evolution, Adaptation, Cactophilic *Drosophila*

## Abstract

**Background:**

Adaptation to new hosts in phytophagous insects often involves mechanisms of host recognition by genes of sensory pathways. Most often the molecular evolution of sensory genes has been explained in the context of the birth-and-death model. The role of positive selection is less understood, especially associated with host adaptation and specialization. Here we aim to contribute evidence for this latter hypothesis by considering the case of *Drosophila mojavensis*, a species with an evolutionary history shaped by multiple host shifts in a relatively short time scale, and its generalist sister species, *D. arizonae*.

**Results:**

We used a phylogenetic and population genetic analysis framework to test for positive selection in a subset of four chemoreceptor genes, one gustatory receptor (*Gr*) and three odorant receptors (*Or*), for which their expression has been previously associated with host shifts. We found strong evidence of positive selection at several amino acid sites in all genes investigated, most of which exhibited changes predicted to cause functional effects in these transmembrane proteins. A significant portion of the sites identified as evolving positively were largely found in the cytoplasmic region, although a few were also present in the extracellular domains.

**Conclusions:**

The pattern of substitution observed suggests that some of these changes likely had an effect on signal transduction as well as odorant recognition and protein-protein interactions. These findings support the role of positive selection in shaping the pattern of variation at chemosensory receptors, both during the specialization onto one or a few related hosts, but as well as during the evolution and adaptation of generalist species into utilizing several hosts.

**Electronic supplementary material:**

The online version of this article (10.1186/s12862-018-1250-x) contains supplementary material, which is available to authorized users.

## Background

Studying host adaptation is critical to understanding the ecological and evolutionary history of phytophagous insects [1–5]. Host shifts represent a distinct and sometimes novel set of complex challenges since new host plants can differ in nutritional content, chemical composition, microorganisms and defensive quality [6–8]. The use of novel resources when shifting to a new host can therefore affect survival of larva and adult stages, fecundity of feeding females as well as behavioral patterns of oviposition and mating, causing ultimately substantial impact on fitness. This has led to the hypothesis that in addition to genes involved in performance relative to a new environment, adaptation to new hosts often involves mechanisms of host preference [8–10]. Given their role in odorant and gustatory sensory and ultimately in the discrimination of suitable hosts, chemoreceptors have been the focus of several studies examining the evolutionary mechanisms underlying host adaptation [11–17].

Chemical stimuli recognition occurs through odorant (*Or*) and gustatory receptor (*Gr*) proteins, converting volatile and soluble chemicals from the environment into electrical outputs through nerve impulses [18, 19]. In insects, these proteins have seven trans-membrane domains located on the dendritic membranes of sensory neurons housed in sensilla of receptor organs, with a reverse orientation when compared to mammalian topology (COOH-tail extracellular) [20, 21]. While the number of genes in these families seems to be largely stable across *Drosophila* evolution, there have been constant gene loss and gain events through the birth-and-death model in insects [22–28] where divergence arises from the selection of beneficial mutations between paralogous genes [29]. This mode of evolution, as well as the clustered organization of these genes has provided strong support to the hypothesis that these loci play a critical role in ecological shifts and specialization [9, 11–17, 22, 23]. In particular, the ecological specialization observed within *Drosophila* has been suggested to have contributed to the higher pseudogenization levels observed in sensory genes [14, 15]. However, the analysis of 12 *Drosophila* species genomes [30], has shown that genome size or endemism could also predict pseudogenization rates, but the few specialist species compared and the lack of evolutionary data over short time scales has prevented efforts to disentangle these causes [31].

The dramatic heterogeneity in family size and divergence among chemosensory gene paralogs suggest that these gene families have evolved rapidly. However, the pattern of variation and divergence at a majority of genes are more consistent with purifying selection and only a small portion of genes have shown evidence of positive selection, some of which have been linked to specialization [14, 15, 29, 31]. Thus, specialist species such as *D. sechellia* and *D. erecta* have shown higher *K*_*a*_*/K*_*s*_ ratios when compared with their closest generalists species [15]. The majority of amino acid sites showing signatures of positive selection in these families have been found in the cytoplasmic domain, suggesting that selection changes are associated with signal transduction rather than the detection of odorant molecules [32]. To date most comparisons have involved a few highly divergent species; here we examine the case of the cactophilic local specialist *D. mojavensis* and its generalist sister species *D. arizonae* to investigate the role of positive selection in four candidate chemoreceptor genes (one *Gr* and three *Or*). These species have adapted to develop and feed on necrotic cacti, showing partially overlapping distributions in xeric regions of southwestern United States and northwestern Mexico [33, 34]. The four candidate loci examined here (*Or67c*, *Or83c1*, *Or83c2* and *Gr63a*) have shown a pattern of differential expression associated with host shifts suggesting their role in host preference, presumably by detecting volatiles associated with either the presence of nutrients or toxins produced in rotting cacti [35–37]. *Or67c* and *Gr63a* are known to play a role in detecting the by-products of alcoholic fermentation as well as CO_2_ [38, 39], while *Or83c1* and *Or83c2* have been suggested to play role in food detection [38, 40].

**Fig. 1:**
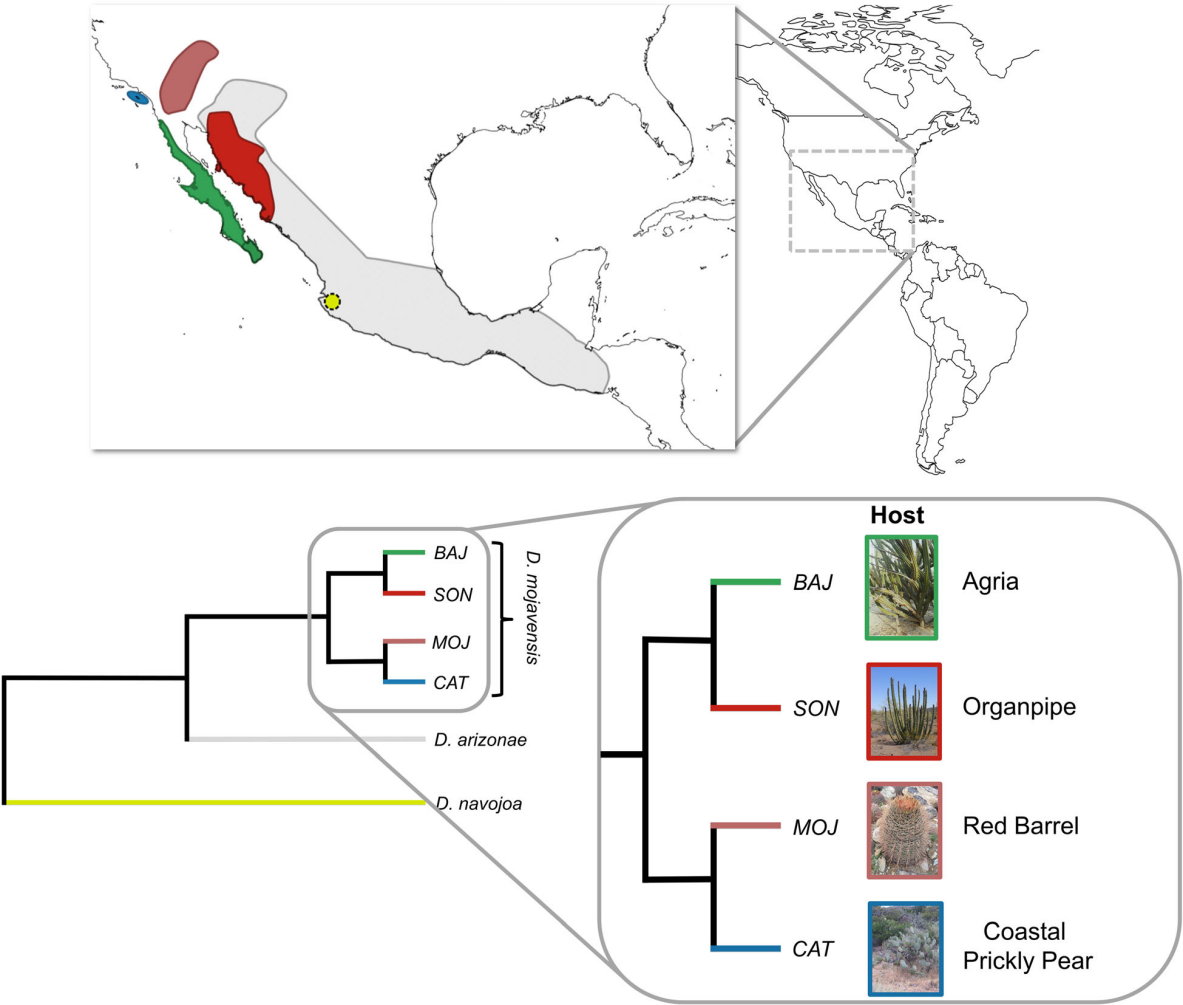
Map showing geographic distributions and evolutionary relationships of *D. mojavensis* host populations, the cactus generalist *D. arizonae* and *D. navojoa* (*Opuntia sp.* breeder*)*. *D. mojavensis* lines were sampled from each population: Baja California (*BAJ*), Sonora Desert (*SON*), Mojave Desert (*MOJ*) and Santa Catalina Island (*CAT*) (cactus hosts are indicated). *D. arizonae* lines were sampled from the Sonora Desert population while the sole *D. navojoa* line was sampled from Jalisco, Mexico. Map was made using public domain vector and raster data from Natural Earth (www.naturalearthdata.com) and then modified to highlight species distributions

*Drosophila mojavensis* offers a unique case to study host adaptation since its biogeographical history has been shaped by multiple host shifts in a relatively short period of time [41]. Since its divergence from its sister species *D. arizonae*, the *D. mojavensis* lineage has expanded through a rapid specialization process (< 0.5 Mya; [42, 43]) producing four host populations adapted to different cactus species (Fig. 1): agria (*Stenocereus gummosus*) in Baja California; coastal prickly pear (*Opuntia littoralis*) in Santa Catalina Island; red barrel (*Ferocactus cylindraceus*) in the Mojave Desert and organpipe (*S. thurberi*) in the Sonora Desert population [33, 34]. We investigated patterns of molecular evolution in four chemoreceptor genes across the four host populations of *D. mojavensis* and the generalist *D. arizonae* to test for positive selection using a multiple approach of gene-wide, polymorphic-based, codon-based and functional tests. Then, we analyzed the distribution of sites showing evidence of positive selection on the structure of these trans-membrane proteins in order to test whether selection has been biased towards specific domains and discussed the implication of these results in terms of the role of positive selection in host preference adaptation.

## Results

### Genetic diversity, neutrality tests and divergence

For each locus examined, between 7 and 15 sequences were generated for each of the four *D. mojavensis* populations (Sonora Desert, SON; Santa Catalina Island, CAT; Mojave Desert, MOJ and Baja California, BAJ), as well as between 8 and 12 sequences of *D. arizonae.* After excluding introns, these amplicons had between 1170 bp for *Or83c2* to 1479 bp for *Gr63a* (Table 1). Given the population structure in *D. mojavensis* (Additional file 1: Table S2) [44, 45], we sampled multiple individuals per population. Here we report average values per population of *D. mojavensis* to make them comparable with those in *D. arizonae* (Table 1). Between 12 and 35 polymorphic sites and 6 and 12 different haplotypes were detected across the loci and species examined (Table 1). Diversity estimates in *D. arizonae* often fall between the estimates for individual population in *D. mojavensis* (Additional file 1: Table S2). However, most genetic diversity estimates were higher in *D. arizonae* when compared with average estimates across *D. mojavensis* populations. Thus, haplotype diversity was higher in *D. arizonae* for all genes, genetic diversity for three genes and nucleotide diversity for two of genes analyzed (Table 1). Tajima’s *D* and Fu and Li’s *D* and *F* neutrality tests were not significant for most genes (Table 1). Fu’s *F*_*S*_ statistic was significantly negative for the majority of genes in both species (Table 1). For one of the loci (*Gr63a*) and in one *D. mojavensis* population (MOJ) these tests were significantly positive (Additional file 1: Table S3), indicating a recent bottleneck causing a heterogeneous pattern in the demographic history of this species.

**Table 1:** Descriptive parameters for genetic diversity, neutrality tests and divergence. Results are shown for each gene in *D. mojavensis* and *D. arizonae*. In the case of *D. mojavensis*, genetic diversity and divergence values correspond to the average across the four populations (See Methods for abbreviations)

*Gene/species*	*Gr63a*		*Or67c*		*Or83c1*		*Or83c2*	
	*D. moj*	*D. ari*	*D. moj*	*D. ari*	*D. moj*	*D. ari*	*D. moj*	*D. ari*
*Ns*	1479	1479	1215	1215	1182	1182	1170	1170
*N*	8.5	8	12.5	12	12.2	12	13.7	12
*h*	6.2	8	9	12	8.5	12	8	12
*H*_*d*_	0.929	1.00	0.866	1.00	0.741	1.00	0.831	1.00
*S*	18.2	12	20.5	27	20.7	35	12.2	20
*π*	0.0049	0.0023	0.0052	0.0048	0.0049	0.0073	0.0029	0.0061
*π*_*s*_	0.0180	0.0069	0.0192	0.0159	0.0121	0.0207	0.0094	0.0237
*π*_*ns*_	0.0007	0.0008	0.0010	0.0014	0.0026	0.0031	0.0010	0.0009
*θ*_*W*_	0.0046	0.0031	0.0055	0.0074	0.0059	0.0098	0.0033	0.0057
*θ*_*W s*_	0.0168	0.0107	0.0207	0.0259	0.0152	0.0257	0.0116	0.0210
*θ*_*W ns*_	0.0006	0.0007	0.0010	0.0018	0.0031	0.0048	0.0010	0.0011
*D*_*T*_	0.37	-1.38	-0.41	-1.59	-0.27	-1.15	-0.04	0.36
*D*	0.43	-1.93	-0.01	**-2.60**	-0.65	-1.79	-0.37	0.37
*F*	0.50	-2.21	-0.15	**-2.77**	-0.70	-1.96	-0.35	0.46
*Fs* ^*a*^	**(+)**	**-5.21**	**(-)**	**-7.41**	**(-)**	**-5.52**	**(-)**	**-6.35**
*Φ*_*ST*_	**0.26**	na	**0.35**	na	**0.34**	na	**0.59**	na
*Ka/Ks*	0.036	0.038	0.059	0.066	0.38	0.30	0.27	0.36

Significant and marginally significant values after *FDR* correction are in bold

^a^In the case of *D. mojavensis* the significance (negative or positive) is indicated when at least one population was significant following Additional file 1: Table S3

*D. moj*: *D. mojavensis*

*D. ari*: *D. arizonae*

*π*_*s*_: Nucleotide diversity for synonymous sites

*π*_*ns*_: Nucleotide diversity for nonsynonymous sites

*θ*_*W*_: Total theta Watterson

*θ*_*W s*_: Theta Watterson for synonymous sites

*θ*_*W ns*_: Theta Watterson for nonsynonymous sites

The genetic divergence assessed by comparing the number of synonymous (*Ks*) and nonsynonymous (*Ka*) substitutions ratio (*Ka*/*Ks*) with respect to the *D. navojoa* outgroup was often larger in *D. arizonae* for all loci with the exception of *Or83c1* (Table 1). The genetic structure as assessed by *Φ*_*ST*_ indicated the existence of significant population structure in *D. mojavensis*, with *Φ*_*ST*_ values ranging between 0.26 and 0.59 (Table 1). Population structure was highly significant for all genes as well as the majority of pairwise comparisons between *D. mojavensis* populations (Additional file 1: Table S4).

### Evidence of positive selection

**Fig. 2:**
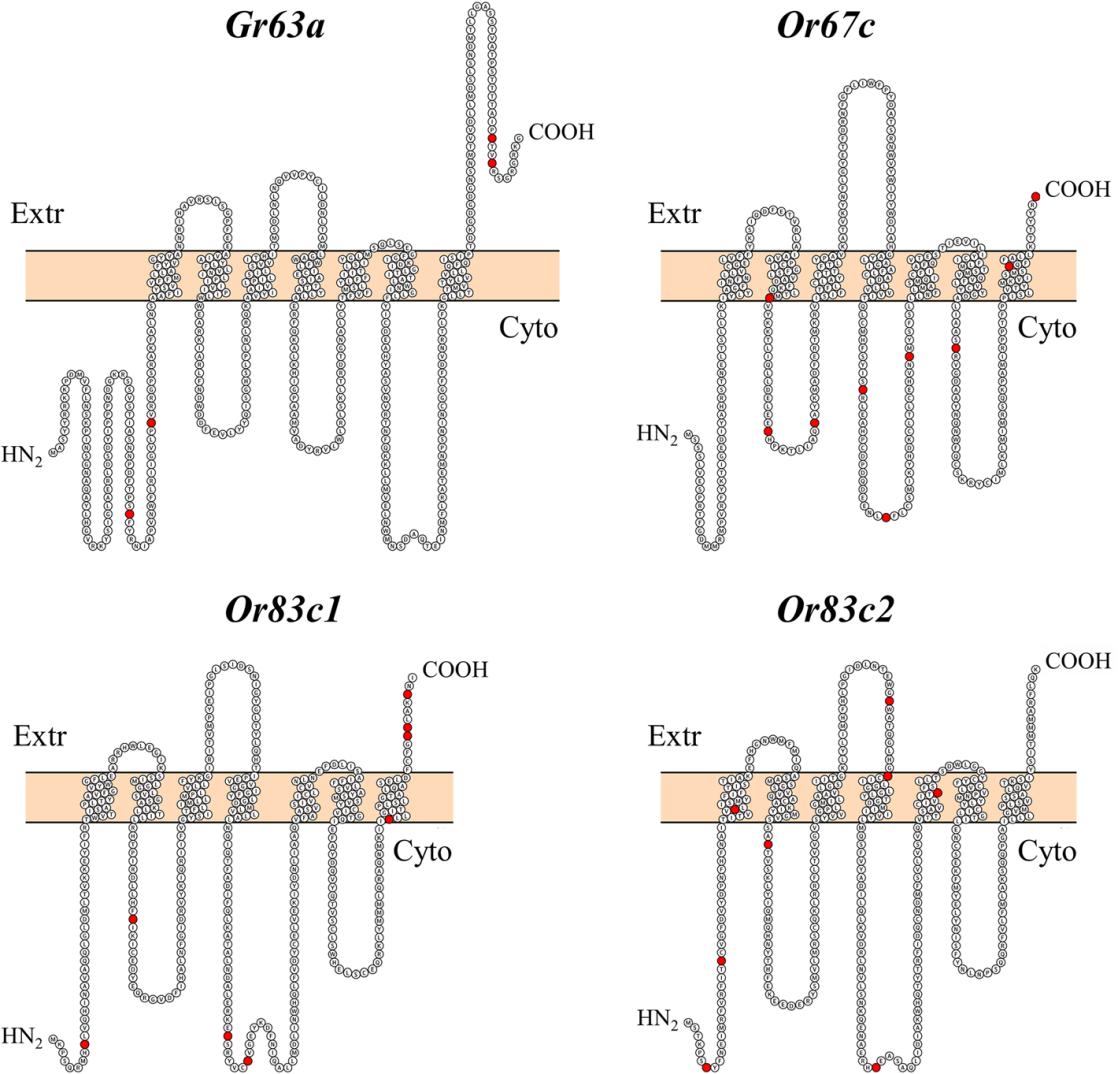
Predicted topology of chemoreceptor proteins showing seven transmembrane domains and a cytoplasmic NH_2_-tail. Positively selected sites are highlighted in red. Topology was predicted by the TMHMM software

All genes showed significant signatures of positive selection in at least one of the gene-wide (Table 2), codon-based and polymorphism-based (Table 3) tests performed. *Codeml* consistently showed all genes evolving under positive selection, ie. LRT favored the M8 selection model (Table 2). The genes *Or67c* and *Or83c2* were also significant in one (*PARRIS*) and three (*PARRIS, BSR*, *aBSREL*) of the additional gene-wide selection tests performed respectively, as well as the McDonald-Kreitman test (Table 2). The BSR test identified selection in *Or83c2* for specific branches belonging to the *SON* and *CAT* populations of *D. mojavensis* as well as branches belonging to *D. arizonae* lineage (Table 2). McDonald-Kreitman test was also performed for pairwise comparisons between host populations of *D. mojavensis* and *D. arizonae*, showing signatures of positive selection for *SON* and *BAJ* in *Or67c* and *SON*, *BAJ* and *CAT* in *Or83c2* (Table 2 and Additional file 1: Table S5)*.* Only the gene *Or83c2* showed signatures of positive selection in both the *D. mojavensis* and *D. arizonae* lineage as determined by the McDonald-Kreitman tests and polarizing the fixed sites utilizing the outgroup species *D. navojoa* (Table 2 and Additional file 1: Table S5). Furthermore, all genes showed evidence of positive selection at individual sites in at least three of the codon-based selection tests performed and all tests provided significant values in at least two genes (Table 3). A combined analysis of these tests led us to identify four sites evolving under positive selection for the gene *Gr63a* (using the *codeml*_*BEB*_*, REL, FUBAR* and *PRIME* tests); nine sites in *Or67c* (using the *codeml*_*BEB*_*, SLAC, FEL, REL, MEME*, *FUBAR* and *PRIME* tests); eight sites in *Or83c1* from (using the *codeml*_*BEB*_*, FEL, IFEL, REL, MEME*, *FUBAR* and *PRIME* tests); and eight sites positively selected in the gene *Or83c1* (using the *codeml*_*BEB*_*, SLAC, FEL, IFEL, REL, MEME*, *FUBAR* and *PRIME* tests) (Table 3, Fig. 2)*.* Though we identified several sites evolving under positive selection, we also investigated whether these changes led to changes in amino acid characteristics that might affect protein structure and hence function. Results of the *PRIME* analysis showed that 38% of the candidate sites across the four loci indicated possible substantial changes to protein structure (Table 3)*.*

**Table 2:** Gene-wide tests of positive selection and McDonald-Kreitman test. A summary of *P*-values obtained for selection tests is shown for each gene. Results for McDonald-Kreitman test are shown for the whole gene as well as for each domain (Cytoplasmic, transmembrane or extracellular) (See Methods for abbreviations)

*Gene*	*Codeml*_*M8*_	*PARRIS*	*BUSTED*	*BSR*	*aBSREL*	*MK*_*Total*_	*MK*_*Cyto*_	*MK*_*Trans*_	*MK*_*Extr*_
*Gr63a*	**< 0.001**	0.131	1.000	> 0.05	> 0.05	0.502	1.000	na	0.350
*Or67c*	**< 0.001**	**0.000**	0.629	> 0.05	> 0.05	**0.015** ^**b**^	0.084	1.000	na
*Or83c1*	**< 0.001**	0.280	0.996	> 0.05	> 0.05	0.885	1.000	0.662	1.000
*Or83c2*	**< 0.001**	**0.017**	0.053	**0.014** ^**a**^	**0.040**	**0.001** ^**c**^	**0.011**	0.661	0.109

Significant and marginally significant values after *FDR* correction are in bold

^a^ This test showed positive selection specific to *D. mojavensis* lineage

^b^ This test was also significant for pairwise comparisons using *D. mojavensis* populations (*SON* and *BAJ*) and *D. arizonae* but not with ancestral *D. navojoa* outgroup (Additional file 1: Table S5)

^c^ This test was also significant for pairwise comparisons using *D. mojavensis* populations (*SON*, *BAJ* and *CAT*) and *D. arizonae* as well as ancestral *D. navojoa* outgroup (Additional file 1: Table S5)

*MK*_*Total*_**:** McDonald-Kreitman test for whole gene

*MK*_*Cyto*_**:** McDonald-Kreitman test for cytoplasmic domain

*MK*_*Trans*_**:** McDonald-Kreitman test for transmembrane domain

*MK*_*Extr*_**:** McDonald-Kreitman test for extracellular domain

**Table 3:** Codon-based tests of positive selection. A summary of *P*-values and posterior probabilities obtained for selection tests are shown for those codons where at least one test resulted with significant value after FDR correction (global α = 0.05). The codon location in domains (*Cyto, trans or extr*) is indicated for each codon under positive selection. Biochemical properties significantly associated with amino acid sites under selection are indicated for *PRIME* test (See Methods for abbreviations)

*Gene/Codon*	Domain	*codeml*_*BEB*_	*SLAC*	*FEL*	*IFEL*	*REL*	*MEME*	*FUBAR*	*PRIME*
*Gr63a*									
73	*Cyto*	0.571	0.471	0.369	> 0.050	**0.980**	0.227	0.732	> 0.050
93	*Cyto*	< 0.500	0.748	0.642	> 0.050	**0.955**	0.487	0.650	> 0.050
482	*Extr*	**0.977**	0.189	0.184	> 0.050	**0.990**	0.078	**0.941**	*V*
485	*Extr*	**0.906**	0.428	0.434	> 0.050	**0.986**	0.139	**0.906**	*H*
*Or67c*									
100	*Trans*	< 0.500	0.132	0.092	> 0.050	**0.933**	0.141	0.648	> 0.050
116	*Cyto*	< 0.500	0.982	0.815	> 0.050	**0.807**	0.583	0.401	> 0.050
125	*Cyto*	< 0.500	0.752	0.529	> 0.050	**0.866**	0.449	0.467	> 0.050
236	*Cyto*	< 0.500	0.199	0.348	> 0.050	**0.901**	**0.023**	0.653	*S*
253	*Cyto*	**0.998**	0.237	**0.029**	> 0.050	**0.976**	0.058	**0.906**	*C, VR*
274	*Cyto*	< 0.500	0.434	0.424	> 0.050	**0.885**	0.388	0.623	> 0.050
335	*Cyto*	< 0.500	0.850	0.965	> 0.050	**0.778**	0.664	0.451	> 0.050
391	*Trans*	**1.000**	**0.025**	**0.000**	> 0.050	**1.000**	**0.000**	**1.000**	*S*
404	*Extr*	**1.000**	0.434	0.268	> 0.050	**0.911**	0.271	0.621	> 0.050
*Or83c1*									
9	*Cyto*	0.764	0.411	0.155	> 0.050	**0.975**	0.133	0.712	> 0.050
99	*Cyto*	**1.000**	0.075	**0.004**	**0.026**	**1.000**	**0.001**	**0.999**	> 0.050
230	*Cyto*	**1.000**	0.088	0.145	> 0.050	**0.999**	0.121	**0.973**	*P, R, I*
236	*Cyto*	**1.000**	0.136	0.105	> 0.050	**1.000**	0.131	**0.972**	*H*
362	*Trans*	0.536	0.451	0.136	> 0.050	**0.976**	0.151	0.727	*> 0.050*
386	*Extr*	**0.999**	0.330	0.051	> 0.050	**0.996**	**0.033**	**0.971**	*H*
387	*Extr*	**1.000**	0.089	0.024	> 0.050	**1.000**	**0.036**	**0.992**	> 0.050
391	*Extr*	**0.965**	0.386	0.070	> 0.050	**0.986**	0.066	**0.926**	> 0.050
*Or83c2*									
7	*Cyto*	0.712	0.459	0.203	> 0.050	**0.972**	0.229	0.828	> 0.050
21	*Cyto*	< 0.500	0.459	0.285	> 0.050	**0.959**	0.064	0.794	> 0.050
43	*Trans*	**1.000**	**0.007**	**0.003**	**0.018**	**1.000**	**0.005**	**0.999**	*C*
92	*Cyto*	0.773	0.572	0.518	> 0.050	0.855	**0.000**	0.644	*C, RP*
178	*Extr*	< 0.500	0.478	0.271	> 0.050	0.720	**0.029**	0.701	> 0.050
187	*Trans*	**0.957**	0.228	0.064	**0.019**	**0.998**	0.085	**0.947**	> 0.050
238	*Cyto*	**1.000**	0.496	0.205	> 0.050	**0.986**	**0.047**	**0.930**	> 0.050
284	*Trans*	0.845	0.176	0.063	> 0.050	**0.992**	0.07	**0.984**	> 0.050

Significant and marginally significant values after *FDR* correction are in bold

Since the performance of selection tests based on phylogenetic comparisons could be affected by recombination increasing the number of false positively selected sites [46–48], we tested for the presence of recombination breakpoints and found evidence for such events in *Or83c1* and *Or83c2*. Therefore, we also performed the test of selection on these two loci within each linkage block. After controlling for recombination, two sites in *Or83c1* and four sites in *Or83c2* continued to indicate a pattern of positive selection. The remaining sites did not pass the threshold of the Bayes factors (BF > 100) in the *REL* test, but posterior probabilities remained over 0.8. Suggesting that, although the power of the test is decreased since blocks are analyzed instead of genes, a similar selective history affected the pattern of variation at these sites as well. For this reason and because the phylogenetic relationships among the three species did not change between partitions, suggesting that the assumptions of selection tests are not violated, only the results for the gene as a whole without partitions were further analyzed.

### Signatures of selection among gene domains

**Fig. 3:**
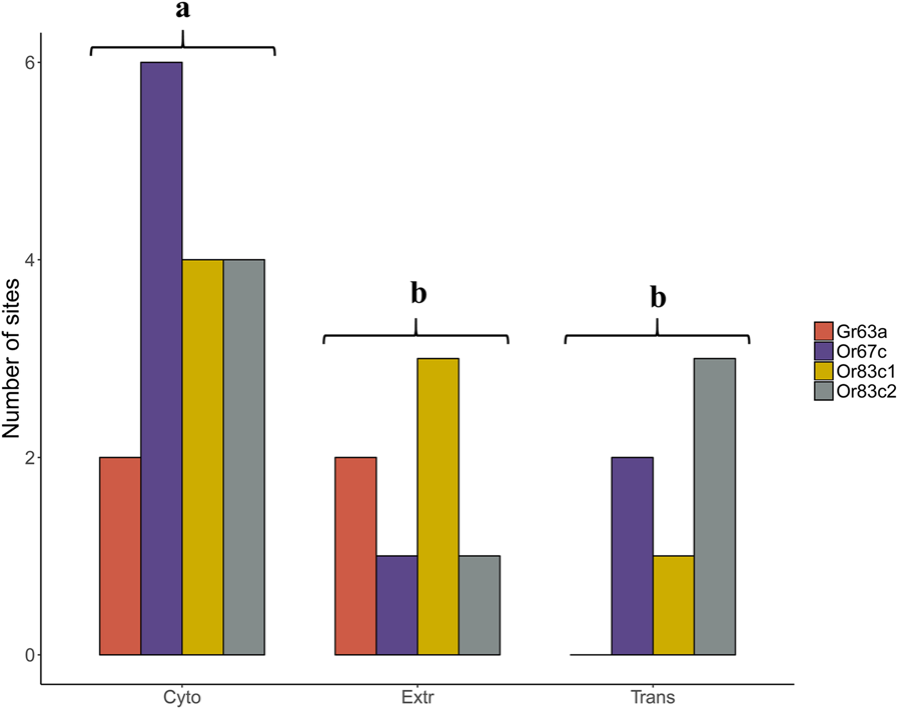
Distribution of the sites showing evidence of positive selection by protein domains in each chemoreceptor gene: Cytoplasmic (Cyto), Extracellular (Extr) and Transmembrane (Trans). The number of sites under positive selection was significantly higher in the cytoplasmic region (Tukey test for multiple comparisons; *p* < 0.05)

Given the pattern observed at these loci suggesting positive selection between *D. mojavensis* and *D. arizonae*, we further tested whether selection had occurred within specific domains of these transmembrane proteins. Approximately 55% of the sites under positive selection are located in the cytoplasmic domain while only 24 and 21% are in the extracellular and transmembrane domains, respectively (Fig. 2). We assessed the distribution of selected sites across the protein domains of all four loci through a *GLM* model (Table 4). We found no significant gene effect, but a significant domain effect (Table 4), with the cytoplasmic domain enriched for positively selected sites when compared with the transmembrane and extracellular domains according to multiple comparisons (Fig. 3). We additionally tested for such structural heterogeneity in the level of selection by running the McDonald-Kreitman independently for each domain of each gene. These tests showed significant evidence of positive selection in the cytoplasmic domain for *Or83c2* and marginally for *Or67c*, while no significant effects for the transmembrane and extracellular domains (Table 2). The proportion of sites under positive selection was just marginally significant when accounting for the domain size in the *GLM* model for *Or83c2* and *Or67c* (Table 4), which points to selection also being influenced by domain size. This is expected since, larger domains have a higher number of sites available for selection to act on.

**Table 4:** *ANOVA* of the *GLM* analysis for the number of sites showing evidence of positive selection and the proportion of sites under positive selection (controlling for the number of amino acids within a domain) (See Methods for abbreviations)

*Effect*	*Df*	*Deviance Resid.*	*Df Resid.*	*Dev.*	*P*
*Number of sites under selection*					
Gene	3	8.8x10^-5^	8	0.0005	0.4366
Domain	2	2.7x10^-4^	6	0.0002	**0.0152**
*Proportion of sites under selection*					
Gene	3	0.0102	8	0.0232	0.1913
Domain	2	0.0103	6	0.0129	0.0925

Significant values are in bold

## Discussion

It has been suggested that specialist species not only lose a subset of sensory pathway genes during host specialization, but some of those that are retained undergo periods of positive selection [14, 15]. Here we investigated the role of positive selection in the molecular evolution of four chemoreceptor genes (one *Gr* and three *Or*) during the evolution of *D. mojavensis*, a species that has experienced recent bouts of specialization resulting in a set of four cactus host populations, and its generalist sister *D. arizonae*. We used multiple approaches to test for positive selection including codon-based, gene-wide, polymorphism and functional analyses. All genes investigated showed evidence of positive selection at codon and gene-wide approaches while two loci indicated positive selection when polymorphism information was analyzed. Although most sites at these genes are likely to be evolving under purifying selection, our approach suggests evidence of positive selection shaping the evolution of several amino acid sites involved in molecular and physiological functions, which allowed us to investigate the distribution of candidate sites under positive selection across the domains of these chemosensory receptor proteins.

The level of genetic diversity was higher in *D. arizonae* when compared with the average value across the *D. mojavensis* populations. This pattern is in agreement with previous studies of other nuclear genes in these species [45], which is likely a consequence of a higher overall effective population size in *D. arizonae* [49, 50]. This pattern of variation is however dissimilar to what has been observed from mitochondrial loci [44], which suggests that recent evolutionary forces are shaping nuclear and mitochondrial genes differently [45]. Likewise, the Fu’s *F*_*S*_ was significantly negative at most loci, which is evidence of excess number of alleles [51]. Since we controlled for the detected population structure performing tests per population, this pattern is more likely indicating that both species are undergoing population expansions. Although we did not find such evidence from all neutrality tests, most values were consistently negative and the Fu’s *F*_*S*_ statistic is particularly sensitive to recent changes in population sizes [51]. This expansion in both species has been previously suggested to be associated with a shared biogeographical history in some of the *D. mojavensis* populations (*SON* and *BAJ*) and *D. arizonae.* As suggested by Machado et al. [45], these populations have experienced expansion and contraction events due to Pleistocene glaciation cycles that affected a great part of the biota [52–54]. This is consistent with our results per population since the sampled population of *D. arizonae* is from the Sonoran Desert and only the populations from Sonora and Baja California showed signatures of expansion in *D. mojavensis*. The sole significantly positive value indicates a deficiency of alleles in the Mojave Desert population of *D. mojavensis* at a single locus (*Gr63a*) [51]. Given that demographic changes would be expected to affect the majority of loci, this particular case could possibly be a result of balancing selection. Further evidence is needed to obtain a more complete understanding of what is shaping variation at this locus in this population.

Most of the evidence for the role of positive selection in *Or* and *Gr* genes during host specialization comes from the genome-wide analysis in *Drosophila* species, particularly the case of *D. sechellia* and *D. erecta* [14, 15, 31]. McBride and Arguello [15] found higher *Ka/Ks* ratios in chemoreceptors of these specialist species when compared with their closest generalists, although such ratios could be alternatively explained by relaxed purifying and not necessarily positive selection. Such comparisons have been based on genome-wide studies, where the lack of polymorphic variation and specialization events in short time scales have made the efforts to disentangle these two hypotheses difficult. Gardiner et al. [32] found 20 genes with evidence of functional divergence in a genome-wide study of 12 *Drosophila* genomes, but only six were considered to be under positive selection. This suggests that the number of genes positively selected in these families is often low in insects, however, as suggested by Gardiner et al. [32], the low power of the phylogenetic-based tests should caution one against making broad generalizations. Despite the low *Ka/Ks* values of these genes, suggesting that most sites are evolving under purifying selection, we were able to identify a number of candidate codons under selection, some of which might contribute to significant effects on protein structure and function. These changes may be involved in detection of new odor molecules as well as protein-protein interactions during signal transduction. These findings suggest that the low number of sites under positive selection previously reported in sensory genes (as recognized by Gardiner et al. [32]) may be at least in part due to the low power of the particular selection tests previously utilized and the absence population level polymorphism data.

According to the specialization hypothesis [14], beneficial mutation associated with amino acid changes that improve the recognition capacity of smell and taste receptors should be favored in specialists, resulting in a narrowly tuned receptor [11]. This proposed mechanism of evolution at chemosensory loci might also have shaped the variation of chemosensory loci during the specialization process of the four cactus host populations of *D. mojavensis* [33, 34, 55]. The necroses of each cactus differ in their chemical composition as well as the yeast and bacterial communities on which *D. mojavensis* feeds [56–58]. It has been shown that these populations not only differ in terms of their performance while utilizing different necrotic cacti [59], but also exhibit different behavioral and electrophysiological responses of olfactory organs, which suggests that their peripheral nervous system has been shaped by local ecological differences [59, 60].

Although we provide evidence supporting the hypothesis of positive selection shaping the evolution of amino acid sites involved in molecular functions of sensory genes during the host specialization in *D. mojavensis*, not all the analyses performed allow for the possibility to detect lineage specific changes. Nevertheless, for those analyses addressing specific lineages, the evidence supports the role of positive selection during the evolution of *D. mojavensis*. This was evident from the *BSR* and McDonald-Kreitman tests in *Or83c2.* While *Or67c* was not significant for the McDonald-Kreitman test when compared with *D. navojoa*, both species showed similar fixed differences. In addition, the changes specific to the *D. mojavensis* lineage are supported by strong genetic differentiation among *D. mojavensis* populations as estimated by *Φ*_*ST*_, which are often larger than what has been previously reported for neutral *SSR* markers [61], suggesting divergent selection may be increasing *Φ*_*ST*_ values in coding sequences among *D. mojavensis* populations.

We also found selective changes exclusive to the *D. arizonae* lineage. This was evident from the *Ka/Ks* value using *D. navojoa* as an outgroup, which was often larger in *D. arizonae* as well as the McDonald-Kreitman tests for *Or83c2* and *Or67c* (Table 2). These findings in *D. arizonae* pose an interesting question about the role of positive selection in the evolution of a generalist that has diverged from a specialized species. Cytological and molecular evidence suggests that the *D. mojavensis*/*D. arizonae* lineage diverged from the lineage of the *Opuntia* specialist *D. navojoa* approximately 4 Mya (reviewed in [41]). Hence *D. arizonae* has acquired the ability to use columnar cacti, as well as *Opuntia*, among other hosts. The potential role of positive selection during the acquisition of new hosts in the evolutionary transition between specialist to generalist species may favor mutations associated with amino acid changes involved in the chemical recognition of a wider repertoire of odors, a broadly tuned receptor [11]. Alternatively, the changes observed could be associated with the modulation of odorant binding signals during the early stages of the signal transduction cascade [62, 63].

The majority of the sites under positive selection in the *Or* and *Gr* genes map to the cytoplasmic domain of these transmembrane proteins (Tables 2, 3 and 4). In general, this pattern was unexpected since this domain interacts with secondary messengers involved in signal transduction and was therefore expected to be conserved. Interestingly, this pattern seems to be more common than previously thought, since there has been a previous report of the cytoplasmic domain of chemosensory receptors evolving rapidly [32]. We only analyzed one *Gr*, which prevents us from including gene family as a factor in the *GLM* model. Despite such limitations of the model, the sole *Gr* gene analyzed here showed clear differences in the distribution of candidate sites when compared with *Or* genes. For example, *Gr63a* had the lowest number of candidate sites and was the only gene with the same number of candidate sites in extracellular and cytoplasmic regions, with no evidence of positive selection in the transmembrane domain. Such differences still need to be confirmed by comparing more *Gr* genes, but again, this result agrees with those patterns reported elsewhere [32]. As suggested by Gardiner et al. [32], this probably reflects the functional and molecular differences between *Or* and *Gr* receptors. *Or* genes are associated with volatile recognition while *Gr* recognize soluble ligands, pheromones and particularly for *Gr63a*, levels of CO_2_ [11, 20, 21, 38, 64] . In addition, the olfactory receptors requires the interaction with the universal receptor *Or83b* [11, 20, 21], while *Gr63a* requires interaction with *Gr21a* for its functionality [39, 65]. Such functional and molecular differences are likely to lead to distinct regions of constraint as reflected by the observed differences in the distribution of positively selected sites.

The majority of positively selected sites mapped to IL1 and IL2 loops, which would be expected since these loops are not involved in the dimerization with *Or83b*, the universal co-receptor [32]. *Or67c* was the only gene showing a candidate site in the IL3 domain. This loop interacts with IL3 of *Or83b* during dimerization [64], which make it likely to be under constraint. We also found candidate sites in the COOH-tail for *Or83c1* and *Or67c*, which contrasts with the results of Gardiner et al. [32] since they found no candidate sites in this region for the ten *Or* genes under positive selection among the 12 *Drosophila* species studied. This terminal region is also important for coupling with *Or83b*, being conserved even in highly divergent genes [64].

The higher number of positively selected sites in the cytoplasmic domain suggest that selection has been acting primarily on the transduction of signal captured by chemoreceptors and not only its detection. Nevertheless, after controlling for the number of amino acids per domain, the *GLM* analysis was just marginally significant. Additionally, *Or83c1* and *Or67c* also show divergent selection in the extracellular region (COOH-tail) essential for signal detection [64], suggesting that both signal detection and transduction have been shaped by positive selection during the molecular evolution of these chemoreceptors.

## Conclusions

Local ecological adaptation can shape the evolutionary trajectories of myriads of traits and their respective underlying genetic architecture. Chemosensory pathways, especially those involved in host preference in insects, can be shaped by the selective forces associated with changes in host utilization. We have provided evidence supporting the hypothesis of host specialization as an evolutionary scenario that favors positive selection at specific amino acid sites in genes of sensory pathways. Given that selection has been particularly biased towards cytoplasmic domains at amino acid sites involved in structural and physiological functions, these selective forces appear to be largely shaping the post-odorant binding role of these chemosensory receptors, i.e. signal transduction. Our approach highlights the advantage of using a set of recently diverged species and populations locally adapted to distinct ecological conditions in the understanding of the mechanisms of chemosensory evolution. Furthermore, the incorporation of population level polymorphism data which allowed multiple and powerful approaches for detecting positive selection in an ecological context proved to be highly effective.

## Methods

### Samples

Isofemale lines from all four *D. mojavensis* cactus host populations were utilized in this study: Baja California (*BAJ*), Sonora Desert (*SON*), Mojave Desert (*MOJ*) and Santa Catalina Island (*CAT*) (Fig. 1). Additionally, sequences from a *D. arizonae* population from the Sonora Desert and one line of *D. navojoa* (*Drosophila* Species Stock Center 15081–1374.11), a cactophilic (*Opuntia* spp) species, from Jalisco, Mexico was utilized for comparisons requiring an outgroup. For both *D. mojavensis* and *D. arizonae* approximately 7–15 independent isofemale lines per population/species were sampled. These isofemale lines have been maintained in the laboratory for 60–120 generations and are highly inbred (Fig. 1). All stocks were maintained in 8-dram vials with banana-molasses media [66] in a 25°C incubator on a 14:10 h light:dark cycle.

### Molecular procedures and alignments

DNA samples for each species were amplified via PCR using Apex Taq DNA Polymerase (Genesee Scientific) following manufacturer’s recommendations in a 25 μl final volume, involving 1 μl of DNA and 0.2 μM of each primer. Amplifications were performed using a PCR program of 35 cycles involving 94 °C for 30 s followed by the corresponding annealing temperature for each gene for 30 s (Additional file 1: Table S1), 72 °C for 1 min, and then a final extension at 72 °C for 10 min. PCR products were purified using QIAquick PCR Purification columns (Qiagen) following manufacturer’s recommended procedures and sanger sequenced in both directions (Operon Eurofins). Sequences were visually inspected for quality using Geneious [67] and aligned based on their amino acid sequences using ClustalW and manual verification. Haplotype phase was inferred for each sample using the software PHASE [68–70].

### Genetic diversity, neutrality tests and divergence

The genetic diversity at nucleotide and haplotype levels was estimated for each species through a set of descriptive parameters using the software DNAsp v5 [71], including the average number of sequences (*N*), total number of sites (*N*_*S*_), number of variable sites (*S*), number of haplotypes (*h*), haplotype diversity (*H*_*d*_), nucleotide diversity (*π*) and the average number of nucleotide differences (*θ*_*W*_). In order to infer any deviation from neutral expectations at the population level, genetic structure or drastic changes in population sizes (recent bottlenecks or population expansion), a set of neutrality tests were performed, including Tajima’s *D*_*T*_ [72], *D* and *F* tests [73] and Fu’s *F*_*S*_ statistic [74] using the software DNAsp. All genes were assessed for recombination events using the *GARD* test, a genetic algorithm for recombination detection [75, 76] implemented in the package HyPhy v2 [77].

Divergence between *D. mojavensis* populations and *D. arizonae* was estimated by the number of synonymous (*Ks*) and nonsynonymous (*Ka*) substitutions ratio (*Ka*/*Ks*) with respect to the *D. navojoa* outgroup using the software DNAsp v5 [71]. The genetic structure among *D. mojavensis* populations was estimated through pairwise *Φ*_*ST*_, an analogue version of the Wright’s fixation index *F*_*ST*_ [78, 79] which is estimated by an Analysis of Molecular Variance (AMOVA), taking into account information on the genetic distances among haplotypes as well as their frequencies following Excoffier et al. [80]. Significance was based on 10,000 permutations in the software Arlequin version 3.5.2 [81].

### Phylogenetic inference

Because several of the selection tests we performed require a phylogenetic tree of aligned sequences, each gene was individually analyzed using Bayesian inference in MrBayes [82] after testing for the best evolutionary model inferred in the software jModelTest v0.1.1 [83]. Two independent runs were used from different starting points by a Metropolis-coupled Markov Chain Monte Carlo analysis with four chains, one cold and three incrementally heated (heating parameter = 0.1) for 10 million generations, sampling every 5,000th tree. Both runs were checked for convergence of the Markov chains with the standard deviation of split frequencies being less than 0.001. Parameter estimates were then analyzed in Tracer [84] to ensure that these had reached stable values with adequate mixing and ESSs above 200.

### Assessing positive selection

Signatures of positive selection were assessed based on the nonsynonymous/synonymous substitution ratio (d_N_/d_S_ = ω). When there is no selection, both classes of substitutions are expected to become fixed with the same probability (ω = 1). On the other hand, selection can increase fixation probabilities for nonsynonymous mutations because of selective advantages (Positive selection, ω > 1), or decrease these probabilities due to selective constrains (Purifying / negative selection, ω < 1). A number of methods have been developed for detecting positive selection based on ω ratios at different levels, whole alignment, phylogenetic branches, codons and combinations of those. Therefore, we tested for selection using multiple tests and four approaches: *i*) gene-wide; *ii*) polymorphic-based; *iii*) codon-based; *iv*) functional implications of candidate sites.

i) Gene-wide selection tests were performed in order to detect general signatures of selection at the gene level in the alignments or trees among sequences of the three species, without making any assumption about foreground branches. First, the software *codeml*, implemented in the package *PAML* v4 [85] was used to compare a null model (M7) in which ω is assumed to be beta-distributed among sites and a selection model (M8), where codons are allowed to have an extra category of positively selected sites ω > 1. The significance of these comparisons was assessed using a likelihood-ratio test [86]. We also used the package HyPhy v2 [77] in order perform a set of gene-wide tests for positive selection to complement and provide a comparison with the LRTs in *codeml*. Thus, we used the *PARRIS* test (a partitioning approach for robust inference of selection), which makes maximum likelihood inference of positive selection robust to the presence of recombination. The *BUSTED* test (Branch-site Unrestricted Statistical Test for Episodic Diversification) [87] was used to specifically assesses whether a gene has experienced positive selection on at least one site and one branch given a phylogeny. The *BSR* (Branch-Site Random effects likelihood test) [88] was used to test for episodic diversifying selection detecting linages with ω > 1. The *aBSREL* test (adaptive Branch-Site Random Effects Likelihood), the improved version of the commonly-used “branch-site” models, was used to test if positive selection had occurred on a proportion of branches. *aBSREL* models both site-level and branch-level ω heterogeneity, testing whether a proportion of sites have evolved under positive selection. An FDR correction was performed to account for multiple comparisons across genes for each test.

ii) A polymorphism-based approach was used to test for positive selection comparing the ω ratios within species with those between species using the McDonald-Kreitman test [89]. This test takes advantage of the intraspecific polymorphic variation, since the substitution ω ratios within species are expected to equal those ratios between species under a neutral scenario but differ under selective scenarios. An FDR correction was performed to account for multiple comparisons across genes for each test.

iii) We performed a set of codon-based selection tests for identifying specific sites within genes showing signatures of selection. Bayes Empirical Bayes (*BEB*) [90] was used to estimate the posterior probability of sites under positive selection following the M8 model from *codeml* results. Again, these results were compared with codon-based selection tests available in the package HyPhy, including *SLAC* (Single-Likelihood Ancestor Counting method) [91], *FEL* (Fixed Effects Likelihood method), *IFEL* (Internal Fixed Effects Likelihood), *REL* (Random Effects Likelihood method), *MEME* (Mixed Effects Model of Evolution) [92] and *FUBAR* tests (Fast Unconstrained Bayesian Approximation for inferring selection) [93].

iv) Finally, we investigated whether changes at the amino acid level were associated with altered biochemical properties using the PRIME test (PRoperty Informed Model of Evolution) following Conant-Stadler [94] and Atchley categories [95]. The five predefined amino acid properties proposed by Conant et al. [94] are: chemical composition of the side chain (C), residue polarity (RP), volume of the residue side chain (VR), isoelectric point of the side chain (IC), and hydropathy (H). The five composite properties proposed by Atchley et al. [95] are: polarity index (P), secondary structure factor (S), volume (V), refractivity (R), and isoelectric point (I). Although the method performs multiple tests on each site, *p*-values are reported after Bonferroni correction to control for the number of false positives.

Sites with evidence of positive selection were mapped onto the receptor’s topology predicted with the software *TMHMM* Server v2 (http://www.cbs.dtu.dk/services/TMHMM/) and then diagrams of the 2D receptors structure were created using *PROTTER* [96].

### Signatures of selection among gene domains

We used two approaches in order to investigate whether selection is acting on specific domains in transmembrane receptor genes (cytoplasmic, transmembrane or extracellular). First, the number of sites under positive selection was compared among genes and domains through a Generalized linear model using the R package *GLM* after data normalization using the logarithmic transformation. This analysis was done twice, for raw counts of sites under selection and after accounting for the size of each domain in order to test for the potential effect of domain size on the number of sites showing signatures of selection, since longer domains have higher chance to show sites under positive selection. Additionally, the McDonald-Kreitman test was performed for each domain independently.

## Additional file

Additional file 1: **Table S1.** Primer sequences (Forward and Reverse) for each gene and species. **Table S2.** Descriptive parameters for haplotype, nucleotide diversity and neutrality tests. Results are shown for each gene in each *D. mojavensis* population and *D. arizonae* (See Methods for abbreviations). **Table S3.** Neutrality tests for each gene in each *D. mojavensis* population and *D. arizonae* (See Methods for abbreviations). **Table S4.** Divergence between *D. mojavensis* and *D. arizonae* species and populations within *D. mojavensis. Ka/Ks* ratios and genetic structure estimated by *Φ*_*ST*_ are given for pairwise comparisons between species and populations for each gene (See Methods for abbreviations). **Table S5.** McDonald-Kreitman test for each *D. mojavensis* and *D. arizonae*. A summary of *P*-values is shown for each gene using *D. arizonae* and D. navojoa as an outgroup (See Methods for abbreviations). (DOCX 50 kb)

## Abbreviations

*aBSREL*: adaptive Branch-Site random effects likelihood
*BAJ*: Baja California
*BSR*: Branch-Site random effects likelihood test
*BUSTED*: Branch-site unrestricted statistical test for episodic diversification
C: Chemical composition of the side chain
*CAT*: Santa Catalina Island
*Codeml*_*M8*_: Codeml comparison between M7 and M8 models
*Cyto*: Cytoplasmic
*D*: Fu and Li’s *D* test
*D. ari*: *D. arizonae*
*D. moj*: *D. mojavensis*
*D. nav*: *D. navojoa*
*D*_*T*_: Tajima’s *D* test
*Extr*: Extracellular
*F*: Fu and Li’s *F* test
*F*_*S*_: Fu’s *F*_*S*_ statistic
H: Hydropathy
*h*: Number of haplotypes
*H*_*d*_: Haplotype diversity
I: Isoelectric point
IC: Isoelectric point of the side chain
*Ka/Ks*: Nonsynonymous (*Ka*)/synonymous (*Ks*) ratio
*MK*_*Cyto*_: McDonald-Kreitman test for cytoplasmic domain
*MK*_*Extr*_: McDonald-Kreitman test for extracellular domain
*MK*_*Total*_: McDonald-Kreitman test for whole gene
*MK*_*Trans*_: McDonald-Kreitman test for transmembrane domain
*MOJ*: Mojave Desert
*N*: Average number of sequences
*N*_*S*_: Total number of sites
P: Polarity index
*PARRIS*: Partitioning approach for robust inference of selection
PRIME: PRoperty Informed Model of Evolution
R: Refractivity
RP: Residue polarity
*S*: Number of variable sites
S: Secondary structure factor
*SON*: Sonora Desert
*Trans*: Transmembrane
V: Volume
VR: Volume of the residue side chain
*θ*_*W ns*_: Theta Watterson for nonsynonymous sites
*θ*_*W s*_: Theta Watterson for synonymous sites
*θ*_*W*_: Total theta Watterson
*π*: Total nucleotide diversity
*π*_*ns*_: Nucleotide diversity for nonsynonymous sites
*π*_*s*_: Nucleotide diversity for synonymous sites
*Φ*_*ST*_: Genetic structure index
