# Supplementary material for: Positive selection at sites of chemosensory genes is associated with the recent divergence and local ecological adaptation in cactophilic Drosophila
Source: BMC Evol Biol. 2018 Sep 20;18:144. doi: 10.1186/s12862-018-1250-x (PMC6148956; doi:10.1186/s12862-018-1250-x)
Supplement: Supplementary file 1 — Table S1. Primer sequences (Forward and Reverse) for each gene and species. Table S2. Descriptive parameters for haplotype, nucleotide diversity and neutrality tests. Results are shown for each gene in each D. mojavensis population and D. arizonae (See Methods for abbreviations). Table S3. Neutrality tests for each gene in each D. mojavensis population and D. arizonae (See Methods for abbreviations). Table S4. Divergence between D. mojavensis and D. arizonae species and populations within D. mojavensis. Ka/Ks ratios and genetic structure estimated by ΦST are given for pairwise comparisons between species and populations for each gene (See Methods for abbreviations). Table S5. McDonald-Kreitman test for each D. mojavensis and D. arizonae. A summary of P-values is shown for each gene using D. arizonae and D. navojoa as an outgroup (See Methods for abbreviations). (DOCX 50 kb) [file 12862_2018_1250_MOESM1_ESM.docx]

**Additional file 1**

**Table S1.** Primer sequences (Forward and Reverse) for each gene and species.

| ***Primer set*** | ***Forward (5' - 3')*** | ***Reverse (5' - 3')*** | ***Tm(°C)*** |
| --- | --- | --- | --- |
|  | |  |  |
| ***D. mojavensis and D. arizonae*** | |  |  |
|  |  |  |  |
| *Gr63a set1* | 98F: AAGAAACAAATAGAGGGTGAGG | 907R: TTCAAGGCCTTCTGGAAAC | 60 |
| *Gr63a set2* | 803F: GGTGGTGCCGTATTGTATATT | 1957R: GCTATTCCCATTGGCTAGTT | 60 |
|  |  |  |  |
| *Or67c set1* | 196F: ACAGCTCCAAGGGCTAAT | 678R: CCGGAAATGTAGCTGAAGTG | 61 |
| *Or67c set2* | 525F: CGCAACTTTGGCTTTCTC | 1527R: CTCGTTCAGCCGCTATT | 59 |
|  |  |  |  |
| *Or83c1 set1* | 231F: GTAGGCTTTGCTGTCCTG | 659R: CGAGTGCGTCGTTCAAT | 60 |
| *Or83c1 set2* | 456F: AAGGGCATACGCATCAC | 1421R: GAATGCAAACCAGCATCAG | 59 |
|  |  |  |  |
| *Or83c2 set1* | 107F: GCCAAATGGCGGCTATAA | 770R: GCGGACTCGGTCTCTATAC | 60 |
| *Or83c2 set2* | 611F: TGCCACTTGGCAATGTT | 1561R: GATTGCTCATTAAACTGTCACG | 60 |
|  |  |  |  |
| ***D. navojoa*** | |  |  |
|  |  |  |  |
| *Gr63a set1* | n91F: AATAGAGGGCGAGGTTAAATG | 405R: CACCGCTAGCAGCATAAA | 61 |
| *Gr63a set2* | 290F: CGCTCCGGTTAATTGGTTTC | 830R: GCCCAGCATAGCTGTCA | 62 |
| *Gr63a set3* | 717F: CTACATTGCCATCGTGCT | 1439R: ACCGTCATATTCGAGTTGC | 60 |
| *Gr63a set4* | 2707F: GCCAGAAGGCGGTCTATATT | 2881R: GCTATTCTAATGTTGTTTATCT | 57 |
|  |  |  |  |
| *Or67c set1* | 1930F: FCGGTGCAGCCCATTAGACAATA | 299R: RCCATGGTGAGCTGCTTGAAGT | 67 |
| *Or67c set2* | 19F: AGAGTCCTCGCACCTTC | 730R: CTCGTCCTGATCTGGGT | 59 |
| *Or67c set1* | 619F: TACTTGGCCGGCATTGCATTC | 1153R: CATGTAGGTCACCAGCGAAATG | 69 |
| *Or67c set2* | 1085F: TCTGCGTGTGGGCGATG | 1546R: GTGCGCTGCTCTATGTGTTTG | 66 |
|  |  |  |  |
| *Or83c1 set1* | 1935F: CAACAGCAATTAGCGTTCTCGG | 883R: CTCAACGAATAGCTGATGCCAGT | 66 |
| *Or83c1 set2* | 622F: GGACCTATGCCGACATTT | 1321R CTTGCGATGCCTCATCAT | 61 |
|  |  |  |  |
| *Or83c2 set1* | 1193F: GCCAAATGGCGGCTATAA | 444R: GCGGACTCGGTCTCTATAC | 61 |
| *Or83c2 set2* | 744F: GTATAGGTCTGATGCCACTTG | 41R: GCTTTGTGCGCTTCATAAC | 60 |
| *Or83c2 set2* | F: AAGTCGTGAGAATGACCAAAGCTG | 110R: ACGGCACTGAAGTGAAAGTTCGGA | 67 |

**Table S2.** Descriptive parameters for haplotype, nucleotide diversity and neutrality tests. Results are shown for each gene in each *D. mojavensis* population and *D. arizonae* (See methods for abbreviations).

| ***Gene/species*** | ***N*** | ***h*** | ***H_d_*** | ***S*** | ***π*** | ***π _s_*** | ***π_ns_*** | ***θ_W_*** | ***θ_W s_*** | ***θ_W ns_*** |
| --- | --- | --- | --- | --- | --- | --- | --- | --- | --- | --- |
|  |  |  |  |  |  |  |  |  |  |  |
| ***Gr63a (NS = 1479)*** |  |  |  |  |  |  |  |  |  |  |
| *D. mojavensis* | 24 | 17 | 0.953 | 37 | 0.0049 | 0.0179 | 0.0006 | 0.0067 | n.a. | n.a. |
| *BAJ* | 8 | 8 | 1.000 | 17 | 0.0045 | 0.0171 | 0.0005 | 0.0044 | n.a. | n.a. |
| *SON* | 9 | 7 | 0.944 | 24 | 0.0046 | 0.0175 | 0.0004 | 0.0060 | 0.0224 | 0.0007 |
| *CAT* | 7 | 5 | 0.905 | 5 | 0.0015 | 0.0063 | 0.0000 | 0.0014 | 0.0056 | 0.0000 |
| *MOJ* | 10 | 5 | 0.867 | 27 | 0.0090 | 0.0313 | 0.0018 | 0.0064 | 0.0224 | 0.0013 |
| *D. arizonae* | 8 | 8 | 1.000 | 12 | 0.0023 | 0.0069 | 0.0008 | 0.0031 | 0.0107 | 0.0007 |
|  |  |  |  |  |  |  |  |  |  |  |
| ***Or67c (NS = 1215)*** |  |  |  |  |  |  |  |  |  |  |
| *D. mojavensis* | 50 | 36 | 0.969 | 52 | 0.0074 | 0.0278 | 0.0013 | 0.0098 | 0.0368 | 0.0017 |
| *BAJ* | 12 | 12 | 1.000 | 33 | 0.0084 | 0.0323 | 0.0012 | 0.0090 | 0.0355 | 0.0011 |
| *SON* | 13 | 13 | 1.000 | 25 | 0.0068 | 0.0265 | 0.0009 | 0.0066 | 0.0254 | 0.0010 |
| *CAT* | 12 | 5 | 0.667 | 12 | 0.0023 | 0.0057 | 0.0013 | 0.0033 | 0.0095 | 0.0014 |
| *MOJ* | 13 | 6 | 0.795 | 12 | 0.0033 | 0.0123 | 0.0006 | 0.0032 | 0.0127 | 0.0004 |
| *D. arizonae* | 12 | 12 | 1.000 | 27 | 0.0048 | 0.0159 | 0.0014 | 0.0074 | 0.0259 | 0.0018 |
|  |  |  |  |  |  |  |  |  |  |  |
| ***Or83c1(NS = 1182)*** | |  |  |  |  |  |  |  |  |  |
| *D. mojavensis* | 49 | 34 | 0.966 | 62 | 0.0065 | 0.0170 | 0.0032 | 0.0118 | n.a. | n.a. |
| *BAJ* | 12 | 12 | 1.000 | 39 | 0.0078 | 0.0194 | 0.0041 | 0.0109 | 0.0282 | 0.0055 |
| *SON* | 11 | 11 | 1.000 | 28 | 0.0071 | 0.0157 | 0.0043 | 0.0081 | 0.0182 | 0.0050 |
| *CAT* | 15 | 5 | 0.109 | 4 | 0.0016 | 0.0050 | 0.0005 | 0.0010 | 0.0033 | 0.0003 |
| *MOJ* | 11 | 6 | 0.855 | 12 | 0.0031 | 0.0084 | 0.0015 | 0.0035 | 0.0109 | 0.0015 |
| *D. arizonae* | 12 | 12 | 1.000 | 35 | 0.0073 | 0.0207 | 0.0031 | 0.0098 | 0.0257 | 0.0048 |
|  |  |  |  |  |  |  |  |  |  |  |
| ***Or83c2 (NS = 1170)*** | |  |  |  |  |  |  |  |  |  |
| *D. mojavensis* | 55 | 32 | 0.958 | 41 | 0.0061 | 0.0183 | 0.0025 | 0.0077 | 0.0269 | 0.0022 |
| *BAJ* | 13 | 13 | 1.000 | 27 | 0.0048 | 0.0195 | 0.0005 | 0.0074 | 0.0289 | 0.0011 |
| *SON* | 14 | 6 | 0.604 | 7 | 0.0010 | 0.0032 | 0.0003 | 0.0019 | 0.0071 | 0.0007 |
| *CAT* | 13 | 8 | 0.910 | 7 | 0.0027 | 0.0080 | 0.0012 | 0.0019 | 0.0060 | 0.0007 |
| *MOJ* | 15 | 5 | 0.810 | 8 | 0.0032 | 0.0069 | 0.0021 | 0.0021 | 0.0046 | 0.0014 |
| *D. arizonae* | 12 | 12 | 1.000 | 20 | 0.0061 | 0.0237 | 0.0009 | 0.0057 | 0.0210 | 0.0011 |

*D. moj*: *D. mojavensis*

*D. ari*: *D. arizonae*

*BAJ*: Baja California

*SON*: Sonora Desert

*MOJ*: Mojave Desert

*CAT*: Santa Catalina Island

*π_s_*: Nucleotide diversity for synonymous sites

*π_ns_*: Nucleotide diversity for non-synonymous sites

*θ_W_*: Total theta Watterson

*θ_W s_*: Theta Watterson for synonymous sites

*θ_W ns_*: Theta Watterson for synonymous sites

**Table S3.** Neutrality tests for each gene in each *D. mojavensis* population and *D. arizonae* (See methods for abbreviations).

| ***Gene/species*** | ***D_T_*** | ***D*** | ***F*** | ***Fs*** |
| --- | --- | --- | --- | --- |
|  |  |  |  |  |
| ***Gr63a*** |  |  |  |  |
| *D. mojavensis* |  |  |  |  |
| *BAJ* | 0.12 | 0.44 | 0.43 | -0.54 |
| *SON* | -1.15 | -1.37 | -1.57 | -0.54 |
| *CAT* | 0.59 | 0.76 | 0.85 | -1.26 |
| *MOJ* | 1.90 | **1.88** | **2.27** | **4.69** |
| *D. arizonae* | -1.38 | -1.93 | -2.21 | **-5.21** |
|  |  |  |  |  |
| ***Or67c*** |  |  |  |  |
| *D. mojavensis* |  |  |  |  |
| *BAJ* | -0.30 | -0.98 | -0.93 | **-4.87** |
| *SON* | -0.18 | -0.69 | -0.65 | **-6.64** |
| *CAT* | -1.25 | 0.83 | 0.28 | 0.59 |
| *MOJ* | 0.09 | 0.80 | 0.71 | 0.63 |
| *D. arizonae* | -1.59 | **-2.60** | **-2.77** | **-7.41** |
|  |  |  |  |  |
| ***Or83c1*** |  |  |  |  |
| *D. mojavensis* |  |  |  |  |
| *BAJ* | -1.32 | -2.04 | -2.23 | **-5.28** |
| *SON* | -0.60 | -0.98 | -1.07 | **-4.84** |
| *CAT* | 1.56 | 1.18 | 1.51 | -0.03 |
| *MOJ* | -0.74 | -0.78 | -1.00 | -0.03 |
| *D. arizonae* | -1.15 | -1.79 | -1.96 | **-5.52** |
|  |  |  |  |  |
| ***Or83c2*** |  |  |  |  |
| *D. mojavensis* |  |  |  |  |
| *BAJ* | -1.52 | -1.80 | -2.10 | **-8.65** |
| *SON* | **-2.07** | -1.82 | -2.24 | **-2.58** |
| *CAT* | 1.60 | 0.67 | 1.05 | -2.01 |
| *MOJ* | 1.85 | 1.47 | **1.89** | 1.91 |
| *D. arizonae* | 0.36 | 0.37 | 0.46 | **-6.35** |

Significant and marginally significant values after *FDR* correction are shaded gray

**Table S4.** Divergence between *D. mojavensis* and *D. arizonae* species and populations within *D. mojavensis.* *Ka/Ks* ratios and genetic structure estimated by *Φ_ST_* are given for pairwise comparisons between species and populations for each gene (See methods for abbreviations).

| **Comparison** | ***Gr63a*** | | ***Or67c*** | | ***Or83c1*** | | ***Or83c2*** | |
| --- | --- | --- | --- | --- | --- | --- | --- | --- |
|  | *Ka/Ks* | *Φ_ST_* | *Ka/Ks* | *Φ_ST_* | *Ka/Ks* | *Φ_ST_* | *Ka/Ks* | *Φ_ST_* |
| *D. moj - D. ari* | 0.088 | **0.748** | 0.141 | **0.669** | 0.174 | **0.783** | 0.231 | **0.842** |
| *BAJ -SON* | 0.030 | 0.025 | 0.034 | **0.071** | 0.195 | **0.157** | 0.125 | **0.464** |
| *BAJ - CAT* | 0.023 | 0.181 | 0.070 | **0.333** | 0.159 | **0.275** | 0.087 | **0.177** |
| *BAJ - MOJ* | 0.058 | **0.275** | 0.026 | **0.340** | 0.184 | **0.231** | 0.122 | **0.554** |
| *SON - CAT* | 0.012 | **0.255** | 0.063 | **0.416** | 0.175 | **0.468** | 0.163 | **0.627** |
| *SON - MOJ* | 0.047 | **0.260** | 0.018 | **0.435** | 0.155 | **0.416** | 0.145 | **0.779** |
| *CAT - MOJ* | 0.049 | **0.380** | 0.073 | **0.562** | 0.245 | **0.557** | 0.150 | **0.645** |

Significant and marginally significant values after *FDR* correction are shaded gray

*D. moj*: *D. mojavensis*

*D. ari*: *D. arizonae*

*BAJ*: Baja California

*SON*: Sonora Desert

*MOJ*: Mojave Desert

*CAT*: Santa Catalina Island

**Table S5.** McDonald-Kreitman test for each *D. mojavensis* and *D. arizonae*. A summary of *P*-values is shown for each gene using *D. arizonae* and D. navojoa as an outgroup (See methods for abbreviations).

| **Species / outgroup** | ***Gr63a*** | | ***Or67c*** | | ***Or83c1*** | | ***Or83c2*** | |
| --- | --- | --- | --- | --- | --- | --- | --- | --- |
|  | *D. ari* | *D. nav* | *D. ari* | *D. nav* | *D. ari* | *D. nav* | *D. ari* | *D. nav* |
| *D. moj* | 0.502 | 0.783 | **0.015** | 0.519 | 0.885 | 0.266 | **0.001** | **0.004** |
| *D. ari* | na | 0.487 | na | 0.862 | na | 0.266 | na | **0.000** |
| *SON* | 0.990 | 0.960 | **0.009** | 0.443 | 0.667 | 0.385 | **0.005** | 0.217 |
| *CAT* | 0.797 | 0.506 | 0.498 | 0.159 | 0.741 | 0.226 | **0.007** | 0.357 |
| *MOJ* | 0.940 | 0.473 | **0.081** | 0.451 | 0.795 | 0.101 | **0.061** | 0.774 |
| *BAJ* | 0.207 | 0.188 | **0.023** | 0.286 | 1.000 | 0.113 | **0.000** | **0.000** |

Significant and marginally significant values after *FDR* correction are shaded gray

*D. moj*: *D. mojavensis*

*D. ari*: *D. arizonae*

*D. nav: D. navojoa*

*BAJ*: Baja California

*SON*: Sonora Desert

*MOJ*: Mojave Desert

*CAT*: Santa Catalina Island
